# A Great Extension Scheme at Cardiff: Colonel Bruce Vaughan's Enterprise and Success

**Published:** 1916-01-22

**Authors:** 


### A GREAT EXTENSION SCHEME AT CARDIFF.

### Colonel Bruce Vaughan's Enterprise and Success.

At the meeting of the board of management of King Edward VII.'s Hospital, Cardiff, held last week, a large and important extension scheme was put forward, which led a member of the medical staff—Major Ewen Maclean—to describe the occasion as an epoch-making one. He declared that the distinction already gained by the hospital was only the distinction of a stage, and it was a blessing that they had among them in Colonel Bruce Vaughan "a man of vision," who would not rest satisfied with the past, but looked forward to the provision of what was still imperative for the needs of the community.

The scheme which Colonel Bruce Vaughan submitted concerned the immediate needs of the hospital, in an order of urgency, which he described as follows:

1. An extension of the children's hospital.
2. The addition of beds for eye cases.
3. The completion of the maternity department.
4. The provision of an orthopædic department.
5. The provision of beds for male and female tuberculosis patients for the purposes of observation and treatment.
6. The provision of extra surgical beds so as to reduce the waiting-list.
7. An additional operating theatre.
8. The extension of the board room, with separate rooms for study for the resident medical officers, and, lastly, a chapel.

In a speech which won unanimous support, Colonel Bruce Vaughan submitted an approximate estimate of the proposed extensions.

The table reproduced herewith cannot fail to prove of interest to hospital people. It gives also the cost of endowment, and Colonel Vaughan subsequently mentioned the only alternative by estimating the additional annual income required for the new beds to be £10,570.

The scheme is certainly ambitious; its total estimated cost is £46,506 for building, and, say, £160,000 for endowment. The money for the maternity department has already been given to Colonel Bruce Vaughan by an anonymous donor, and the recent gifts to the hospital from well-known firms at Cardiff Docks (recorded in our issue of 15th inst.) make it evident that it is not an inopportune time, at any rate in Cardiff, to ask for money. Another anonymous donor handed £10,000 to Colonel Vaughan on the 18th inst., so that practically a third of the building fund has been provided in less than a week. The balance should be in the Colonel's hands within a month. Whether the scheme succeeds or fails—and failure is, surely, unthinkable at Cardiff—it can be proceeded with in its entirety or by section, and the sections can be dealt with in their order of urgency. It is hoped that the need for each section may make a special appeal to individual donors, as has already happened in the case of the maternity flat.

| | No. of Beds | Cost to Build £ | Cost to Endow £ |
|---|---|---|---|
| No. 1 New Children's Ward ... ... ... | 15 | 3,050 | 15,750 |
| No. 2 New Children's Ward ... ... ... | 10 | 2,650 | 10,500 |
| No. 1 New Eye Ward ... | 14 | 2,778 | 14,700 |
| No. 2 New Eye Ward ... | 12 | 2,500 | 12,600 |
| New Maternity Flat ... | 18 | 5,000 | 18,900 |
| Orthopædic Ward ... ... | 14 | 2,778 | 14,700 |
| No. 1 Medical Tubercular Ward ... ... ... | 10 | 2,650 | 10,500 |
| No. 2 Medical Tubercular Ward ... ... ... | 8 | 2,200 | 8,400 |
| New Male Surgical Ward... | 20 | 6,500 | 21,000 |
| New Female Surgical Ward | 20 | 6,500 | 21,000 |
| Addition to "Insole" Ward ... ... ... | 10 | 1,400 | 10,500 |
| New Operating Theatre ... | — | 2,500 | — |
| Lifts ... ... ... ... | — | 1,000 | — |
| New Board Room Students' Rooms, and Chapel ... | — | 5,000 | — |
| Total ... ... | 151 | 46,506 | 158,550 |

It is hoped that Colonel Bruce Vaughan's scheme not only may receive the success it deserves, but also that the Treasury may be moved to permit the building of the medical school to be completed, for the entire cost of which Sir William James Thomas has made himself responsible. The delay in completing the buildings is not only disappointing to its promoters, but a positive set-back is feared, and unless the medical school, and all that it means to the life of the hospital, be placed in a position of active usefulness, the hospital's proposed extensions may be robbed of much of their efficiency. The proposals were referred to a committee. It is a remarkable fact that within a week Colonel Bruce Vaughan should have received a third of the sum required to make his proposals immediately effective. All this money was given to the Building Fund, and it is essential that the balance of £30,000 should first be subscribed and completed quickly. Experience shows that Cardiff is prospering, and we hope that not only the Building but the Endowment Funds will be completed in record time.

